# Rapid identification of a major diffusion/perfusion mismatch in distal internal carotid artery or middle cerebral artery ischemic stroke

**DOI:** 10.1186/1471-2377-12-132

**Published:** 2012-11-05

**Authors:** Reza Hakimelahi, Albert J Yoo, Julian He, Lee H Schwamm, Michael H Lev, Pamela W Schaefer, Ramon Gilberto González

**Affiliations:** 1 Neuroradiology Division, Massachusetts General Hospital, Harvard Medical School, Boston, MA, 02114, USA; 2 Department of Neurology, Massachusetts General Hospital, Harvard Medical School, Boston, MA, 02114, USA

## Abstract

**Background:**

We tested the hypothesis that in patients with occlusion of the terminal internal carotid artery and/or the proximal middle cerebral artery, a diffusion abnormality of 70 ml or less is accompanied by a diffusion/perfusion mismatch of at least 100%.

**Methods:**

Sixty-eight consecutive patients with terminal ICA and/or proximal MCA occlusions and who underwent diffusion/perfusion MRI within 24 hours of stroke onset were retrospectively identified. DWI and mean transit time (MTT) volumes were measured. Prospectively, 48 consecutive patients were identified with the same inclusion criteria. DWI and time to peak (TTP) lesion volumes were measured. A large mismatch volume was defined as an MTT or TTP abnormality at least twice the DWI lesion volume.

**Results:**

In the retrospective study, 49 of 68 patients had a DWI lesion volume ≤ 70 ml (mean 20.2 ml; SEM 2.9 ml). A DWI/MTT mismatch of > 100% was observed in all 49 patients (P < .0001). In the prospective study, there were 35/48 patients with DWI volumes ≤ 70 ml (mean 18.7 ml; SEM 3.0 ml). A mismatch > 100% was present in all 35 (P < .0001).

**Conclusions:**

Acute stroke patients with major anterior circulation artery occlusion are exceedingly likely to have a major diffusion/perfusion mismatch if the diffusion lesion volume is 70 ml or less. This suggests that physiology-based patient assessments may be made using only vessel imaging and diffusion MRI as a simple alternative to perfusion imaging.

## Background

Patients with a major ischemic stroke syndrome due to an occlusion of a major anterior circulation artery constitute an important subset of stroke patients. They commonly have severe neurological deficits and are at high risk of having a poor outcome such as death or dependent life. This is because occlusions of the terminal internal carotid artery (ICA) and/or the middle cerebral artery (MCA) interrupt blood flow to the MCA territory, which comprises a large proportion of the cerebral hemisphere that includes much eloquent cortex. The blood flow physiology in this type of stroke has interesting properties due to the collateral circulation provided by pial collaterals from the ipsilateral anterior and posterior cerebral arteries. A robust collateral circulation can preserve a large portion of the MCA territory for long periods. The collateral circulation also makes infarct ‘core’ and the underperfused ‘penumbra’ in the MCA territory dependent variables; if one is small, the other must be large, and vice versa. The presence of a small ‘core’ and large ‘penumbra’ raises the possibility of treatment.

Reperfusion is a possible therapy, but it is necessary to rapidly assess whether successful reperfusion will help the patient significantly or whether the infarct is already too large to make a clinical difference. The diffusion/perfusion mismatch has been suggested as guide for this purpose, but it remains controversial [1,2]. All agree that patients with small regions of infarction and large areas of poorly perfused brain tissue are likely to benefit from reperfusion and those with large infarcts and small areas of uninfarcted poorly perfused brain are poor candidates.

Problems however lie with detection of this information. DWI is accepted as a very good (but not perfect) measure of infarction (Class I, level of evidence A) [3]. Perfusion imaging is more problematic. It is difficult to quantify [3-5]. In patients various parameters – time to peak, mean transit time, area under the curve, etc. – differ [5,6]. Some regions within the underperfused region are severely threatened while decreased perfusion in other areas is minor and readily reversible. The contrast used during perfusion imaging has risks [7]. The procedure prolongs the imaging and it takes time for the results of the analysis of the data to be delivered to the treating physicians. In contrast DWI results are immediately available and relatively simple to interpret.

Because of the properties of the collateral circulation, we hypothesized that, in patients with occlusions of the distal ICA and/or mainstem MCA, the volume of DWI abnormality would be inversely related to the volume of decreased perfusion. Those patients with large DWI lesions would have small residual areas of non-infarcted but underperfused tissue, and those patients with small DWI lesions would have relatively large regions of still at-risk tissue. If this proves correct then MRI with DWI imaging along with MRA or CTA of the supply arteries would prove sufficient for decisions regarding reperfusion. In this study we used a DWI lesion volume of 70 ml as the cut-off.

## Methods

### Patient selection

This study consisted of retrospective and prospective components. Both components were compliant with the Health Insurance Portability and Accountability Act (HIPAA) and were approved by our institutional review board (IRB).

The retrospective study utilized data from a published study by Copen et al. [8], which demonstrated that a clinically significant diffusion/perfusion mismatch is common among acute ischemic stroke patients imaged within 24 hours of stroke onset, and most commonly occurs in patients with a proximal artery occlusion. In that study, all patients who presented to the emergency department (ED) of our hospital from June 2005 through December 2006 with symptoms suggesting an acute stroke syndrome were screened. Inclusion criteria for the present analysis were:

1. MR imaging including DWI scan demonstrating an acute anterior circulation infarct and perfusion-weighted imaging.

2. CT angiography (CTA) or MR angiography (MRA) of the head demonstrating a proximal anterior circulation artery occlusion (i.e. terminal ICA and/or proximal MCA).

3. All imaging was performed before any thrombolytic therapies and within 24 hours of symptom onset.

In the second study, we prospectively identified patients who presented to our hospital ED with stroke symptoms in 2008. Patients were included in this study if they met the same criteria as in the retrospective study. Patients who received intravenous thrombolytic prior to imaging were included if their imaging demonstrated a diffusion/perfusion mismatch consistent with persistent occlusion.

### Imaging acquisition

All imaging studies were requested by ED physicians based on clinical need and were not influenced by these studies.

### Computed tomography angiography

In the retrospective study, CTA was performed from the C6 vertebral body level through the circle of Willis following injection of 100–140 ml of Isovue 61.2 g/100 mL (Bracco Diagnostics, Princeton, NJ) at a rate of 3 ml/s. Imaging was triggered 25 seconds after contrast material injection (40 seconds for patients with atrial fibrillation). Immediately afterward, a second set of images was obtained from the aortic arch to the skull base. The parameters were 2.5-mm slice thickness, 1.25-mm reconstruction interval, 140 kV, 220–250 mA, and 0.75:1 pitch.

In the prospective study, CT angiography was performed from the vertex to the aortic arch following injection of 80–120 ml of Isovue 370 (Bracco Diagnostics, Princeton, NJ) at a rate of 3.5 ml/s. SmartPrep (GE Medical Systems) was used with a region of interest 1 cm below the carina covering the entire lumen of the ascending aorta. Scanning began with a 10-second delay after the region of interest reached 75 HU. The parameters were 1.25-mm slice thickness, 0.625-mm reconstruction interval, 120 kV, 350–800 mA, and 0.516:1 pitch.

### Magnetic resonance imaging

MR imaging was performed on a 1.5 T Signa whole body scanner (GE Medical Systems) with echo-planar capabilities. Axial DWI were obtained using single-shot, spin echo echoplanar imaging with the following parameters: TR 5000 ms; TE 80 to 110 ms; b-value 1000 s/mm2; field of view 22 cm; matrix size 128_128, zero-filled to 256_256, and slice thickness of 5 mm with a 1-mm inter-slice gap. As many slices as needed to cover the entire brain were acquired. For each slice a sequence including an image without diffusion gradients plus high-gradient-factor images in six directions were acquired. This was repeated five times and resulted in 35 images for each slice and a total imaging time of less than 4 minutes. Double inversion pulses were used to help reduce eddy current effects.

The perfusion-weighted imaging (PWI) was a serial gradient echo echoplanar sequence with TR/TE of 1500/40 ms. The field of view, section orientation, slice thickness, and spacing were as specified previously in the DWI sequence. Ten seconds after the beginning of image acquisition, 20 mL gadopentetate dimeglumine 0.5 mol/L (Magnevist; Bayer HealthCare Pharmaceuticals) was injected at a rate of 5 mL/s followed by 20 mL normal saline at the same rate. Sixteen slices were acquired every 1.5 seconds and this was repeated 46 times, which resulted in 69 seconds of imaging time.

MRA images of the head were acquired using a three-dimensional time-of-flight technique with 25° flip angle, TR/TE of 36/6.8 ms, 18-cm field of view, and a 512 _ 512 matrix. One hundred eleven transverse images were reconstructed with a section thickness of 1.4 mm and spacing of 0.7 mm.

### Image processing

In the retrospective study, the mean transit time (MTT) map was calculated using singular value decomposition deconvolution [9] with the arterial input function (AIF) derived from the middle cerebral artery ipsilateral to the infarct. This map was used as its clinical utility has been demonstrated in multiple successful trials [10,11]. We used the time to peak (TTP) map in the prospective study because it is more commonly used. It has been shown to have the highest predictive performance when compared to standard MTT, circular MTT, and time to maximum (Tmax) maps [6]. Image processing usually consumes approximately 5 to 10 minutes; however exact processing time was not available in our studies.

### Post processing image analysis

In the retrospective study, lesions on DWI and MTT maps were outlined visually by a research technologist and were edited by a neuroradiologist (P.W.S.) using a semi-automated commercially available image analysis program (Analyze 7.0; AnalyzeDirect, Overland Park, Kan). These were done blinded to time of stroke onset and the locations of arterial occlusion. In the prospective study, DWI abnormalities were outlined visually by a research fellow and were checked by a neuroradiologist (R.G.G.). After coregistration, PWI lesions were outlined using a TTP delay threshold of greater than four seconds relative to the contralateral hemisphere, which was found to be the best estimate of penumbral tissue in a correlative study of MRI and positron emission tomography [12]. The mean value of a region of interest covering the contralateral MCA territory at the level of the basal ganglia was the reference for identifying tissue with TTP delay beyond the 4 second threshold [12]. Perfusion map artifacts such as those arising within the ventricles were excluded manually. Final outlines were checked by the same neuroradiologist. All analyses were performed using a semi-automated commercially available image analysis program (Analyze 8.0; AnalyzeDirect, Overland Park, Kan) and blinded to the clinical data of the patients and the site of arterial occlusion.

The absolute volume of the diffusion-perfusion mismatch was calculated as the difference between the respective volumes. Areas of DWI abnormalities which were not covered by PWI lesions were added to the total perfusion abnormality volume for mismatch calculation. A mismatch was considered zero if the PWI lesion was smaller than the DWI lesion. Absolute mismatch volume was divided by DWI lesion volume to yield relative mismatch volume in percent, a value that has been used in different clinical trials to recruit patients who may benefit from thrombolytic therapies [10,11].

### Statistical analysis

Patients with DWI lesions ≤ 70 ml were compared to those with lesions > 70 ml for the likelihood of the presence of a 100% diffusion/perfusion mismatch using two-tailed Fisher’s Exact Test. This analysis was repeated using a DWI lesion volume threshold of 100 ml. Two-tailed Spearman’s rank analyses were performed to assess for a significant correlation between time from ictus and DWI lesion volume, and between time from ictus and relative mismatch values. A finding was deemed significant if the P value was <0.05.

## Results

**Figure 1:**
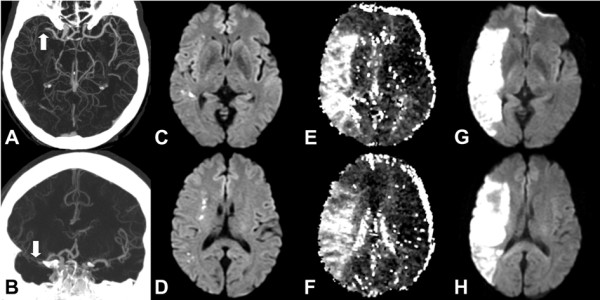
**61 year old male presenting with left facial droop and dysarthria.** Initial imaging studies were performed 6 hours after witnessed onset of speech difficulties. However, patient noted symptoms suggestive of facial weakness 12 hours prior to imaging. **A** &**B**. CTA demonstrates a proximal right MCA occlusion (white arrows). **C** &**D**. Initial DWI shows multiple punctate foci of diffusion abnormality consistent with a small acute infarct. **E** &**F**. Time to peak (TTP) MR perfusion map demonstrates a large volume of underperfused area involving the majority of the right MCA territory and consequently a large diffusion/perfusion mismatch. The patient did not undergo thrombolytic therapy because of the uncertain time between stroke onset and presentation. **G** &**H**. Follow-up MRI shows an infarction involving the majority of the right MCA territory. Whole brain map volume measurements for this patient are: DWI: 2.1 ml; MTT: 117 ml (mismatch: 115.4 ml); TTP: 28 ml (mismatch 25.9 ml).

**Figure 2:**
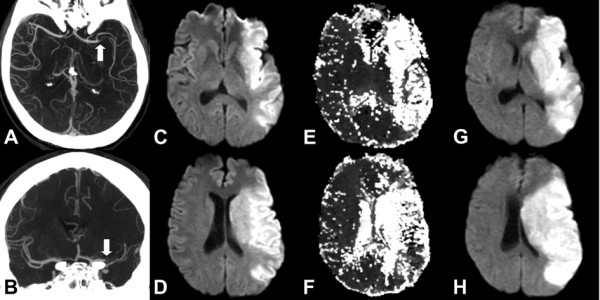
**51 year old male presenting to the ED with right hemiparesis and aphasia.** Imaging was initiated 4 hours after witnessed symptom onset. **A** &**B**. CTA demonstrates occlusion of the proximal left MCA (white arrows). **C** &**D**. Initial DWI shows large abnormality of the left MCA territory. **E** &**F**. MR perfusion time to peak (TTP) maps demonstrates hypoperfusion of similar extent as the diffusion abnormality. **G** &**H**. Follow-up MRI shows no significant enlargement of the initial abnormality. Whole brain map volume measurements for this patient are: DWI: 154 ml; MTT: 211 ml (mismatch: 57 ml); TTP: 241 ml (mismatch 87 ml).

Figures 1 and 2 illustrate the hypothesis that was tested. In both cases, the patients underwent imaging that included CTA followed by diffusion/perfusion MRI. Both patients were found to have proximal anterior circulation artery occlusion. However, one had a small DWI lesion volume (Figure 1) at presentation, while the other (Figure 2) had a very large DWI lesion volume. Our hypothesis predicts that the patient with the small DWI lesion will have a large diffusion/perfusion mismatch, but not the patient with the large DWI abnormality. The predictions were verified.

**Figure 3:**
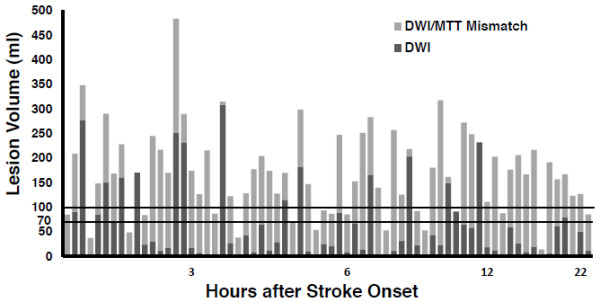
**DWI and mismatch volumes of entire retrospective cohort in order of time after stroke onset.** Abnormal DWI volume of each patient is depicted as a dark gray bar. Light gray bars represent DWI/MTT mismatch. Horizontal lines demarcate volumes of 70 ml and 100 ml. All patients with DWI lesion volume 70 ml or less had at least a 100% mismatch. There was no significant correlation between time from stroke onset and DWI lesion volume or between time and mismatch volume. DWI: Diffusion weighted imaging; MTT: Mean transit time.

There were 68 patients in the retrospective study. Figure 3 is a histogram that displays the DWI and mismatch volumes of the entire cohort. There were 49 (72%) patients with volumes ≤70 ml and 19 (28%) patients with volumes >70 ml. A mismatch of 100% or greater was found in all 49 patients with DWI lesion volume ≤ 70 ml but in only 4 of 19 (21%) patients with a larger DWI abnormality (p < 0.0001) (Table 1). Even with an abnormal DWI volume threshold of 100 ml, 96.4% (53/55) of patients had a mismatch ≥100%. No patient with an abnormal DWI volume >100 ml had a mismatch of 100% or greater (p < 0.0001) (Figure 3).

**Table 1:** Demographic and imaging data of the retrospective cohort

	**All**	**DWI lesion** ≤**70 ml**	**DWI lesion >70 ml**	***P *value**
**Number (%)**	68(100%)	49(72.1%)	19 (27.9%)	0.014
**Age (median; IQR)**	74.2; 27.7	74.2; 24.2	79.9; 42.3	0.554
**Female**	37 (54.4%)	27 (55.1%)	10 (52.6%)	0.930
**DWI Vol. (median; IQR)**	24.3; 72.6	12.4; 23.6	160.7; 126.7	0.000
**Mismatch Vol. (median; IQR)**	94.4; 91.5	99.2; 95.3	67.1; 103.3	0.007
**% Mismatch (median; IQR)**	347.9; 1534.4	1055.2; 1657.3	47.9; 84.9	0.113
**Large mismatch (≥100%)**	53 (77.9%)	49 (100%)	4 (21%)	0.000

*DWI* = diffusion weighted images; *IQR* = interquartile range.

**Figure 4:**
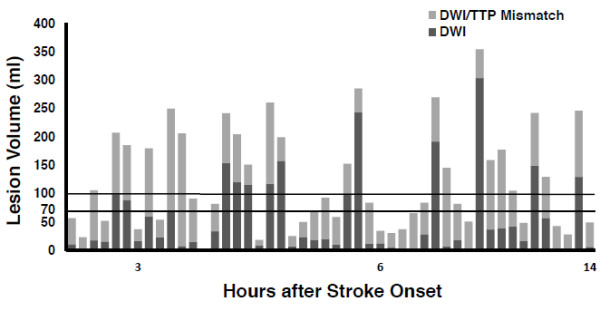
**DWI and mismatch volumes of entire prospective cohort in order of time after stroke onset.** Abnormal DWI volume of each patient is depicted as a dark gray bar. Light gray bars represent the DWI/TTP mismatch. Horizontal lines delineate volumes of 70 ml and 100 ml. All patients with DWI lesion volume of 70 ml or less had at least 100% mismatch volume. There was no significant correlation between time from stroke onset and DWI lesion volume or between time and mismatch volume. DWI: Diffusion weighted imaging; TTP: Time to peak.

In the prospective study, 55 consecutive patients were identified. Seven cases were excluded from evaluation because of poor quality of the perfusion data, motion artifact, and in one case due to stenosis of a contralateral anterior circulation proximal artery. The DWI and mismatch volumes of the entire prospective cohort are shown in Figure 4. There were 35 (72.9%) patients with DWI lesion volumes ≤70 ml, and all had DWI/PWI mismatch ≥100%. Of 13 patients with DWI lesions >70 ml, three (23%) had a mismatch ≥100% (p < 0.0001) (Table 2). Using the 100 ml abnormal DWI volume threshold, 97.4% (37/38) of patients with DWI lesion ≤100 ml demonstrated a mismatch ≥100%, and all had a mismatch ≥50%. Only 1 of the 10 cases with DWI lesion >100 ml had a mismatch of 100% (p < 0.0001) (Figure 4). An analysis performed using MTT data for the prospective cohort had the same result as the analysis using TTP maps (data not shown). Removal of the patients who received IV tPA prior to imaging did not change the results and all 24/32 patients with small DWI lesions had a mismatch ≥100% (p < 0.0001).

**Table 2:** Demographic and imaging data of the prospective cohort

	**All**	**DWI lesion ≤70 ml**	**DWI lesion >70 ml**	***P *value**
**Number (%)**	48 (100%)	35 (72.9%)	13 (27.1%)	0.035
**Age**				
**(median; IQR)**	69.5; 22.3	69; 22.5	71; 20	0.688
**Female**	18 (37.5%)	16 (45.7%)	2 (15.4%)	0.092
**DWI Vol.**				
**(median; IQR)**	19.2; 82.8	15.3; 16.8	129.6; 42.8	0.000
**Mismatch Vol. (median; IQR)**	51.6; 51.7	48.2; 44.1	83.9; 46.7	0.224
**% Mismatch**				
**(median; IQR)**	214.5; 388.1	354.9; 436.7	56.7; 59.2	0.034
**Large mismatch (≥100%)**	38 (79.2%)	35 (100%)	3 (23.1%)	0.000

*DWI* = diffusion weighted images; *IQR* = interquartile range.

Individual patient imaging data and analysis of the retrospective and prospective studies are provided in Tables 3 and 4 respectively. The patient imaging data in Figures 3 and 4 are displayed in order of time after stroke onset. Analysis of the retrospective data set disclosed no significant correlation between time from ictus and DWI lesion volume (p = 0.38) or between time and mismatch volume (p = 0.44). Similarly in the prospective study, there was no significant correlation between time from stroke onset and DWI volume (p = 0.87) or between time and mismatch volume (p = 0.77). In both studies, all patients with small DWI had large mismatches irrespective of their age or gender and there was no effect by these covariates.

**Table 3:** Individual patient imaging data of the retrospective cohort arranged by time after stroke onset

**Hours after stroke**	**DWI volume**	**MTT volume**	**Mismatch volume**	**Mismatch %**
1.4	1	85	83	7049
1.5	90	209	118	131
1.6	277	348	71	26
1.7	2	37	35	1508
1.9	85	148	63	75
2.0	150	290	140	93
2.0	72	168	96	134
2.0	161	228	67	42
2.0	170	14	0	0
2.0	0	49	49	52239
2.2	24	84	60	252
2.3	30	245	215	723
2.4	11	217	206	1898
2.5	17	170	152	871
2.8	252	484	232	92
2.9	232	289	57	25
3.0	17	174	157	912
3.3	7	126	120	1782
3.4	3	216	213	8290
3.4	5	86	81	1663
3.5	308	315	7	2
3.6	27	122	95	349
4.4	2	38	37	2249
4.4	43	128	86	201
4.4	65	204	139	216
4.4	8	177	169	2083
4.5	12	174	162	1321
4.6	29	128	99	347
4.8	115	170	55	48
4.9	0	68	68	49547
5.0	182	299	117	64
5.3	10	147	137	1439
5.6	2	53	51	2780
5.6	25	93	68	276
5.7	20	86	66	326
5.9	89	247	158	178
6.0	8	85	77	976
6.4	67	152	85	126
6.5	14	251	237	1667
6.6	166	283	117	70
6.7	1	140	138	12810
6.7	4	52	49	1356
6.8	11	257	246	2261
7.6	31	125	94	301
7.9	203	218	15	7
8.1	23	92	69	301
8.1	3	52	50	1983
8.2	44	180	136	313
9.0	23	317	295	1307
9.4	149	161	12	8
10.3	91	11	0	0
10.5	65	272	207	319
11.0	58	248	191	330
11.2	232	181	0	0
11.2	18	75	93	513
12.7	12	203	190	1529
13.0	3	87	84	2475
14.0	60	176	116	195
15.2	26	206	180	691
15.8	9	166	158	1818
16.8	19	216	198	1055
17.4	3	14	11	406
18.0	6	191	186	3234
19.5	62	157	95	153
21.1	79	167	87	110
21.2	1	123	122	12052
22.3	50	127	77	153
22.7	11	85	74	658

All volumes are in ml; *DWI* = diffusion weighted images.

**Table 4:** Individual patient imaging data of the prospective cohort arranged by time after stroke onset

**Hours after stroke**	**DWI volume**	**TTP volume**	**Mismatch volume**	**Mismatch %**
0.5	11	57	46	417
1.6	3	18	20	649
1.7	18	106	88	502
2.5	16	52	36	229
2.7	99	208	109	109
3.0	88	186	97	110
3.0	17	37	21	121
3.1	60	180	120	200
4.1	23	54	31	132
4.2	69	250	181	263
4.5	8	206	199	2556
4.5	15	92	76	497
4.7	0	3	3	615
4.7	34	82	48	142
4.8	154	242	88	57
5.0	121	205	84	69
5.0	116	151	36	31
5.1	8	19	10	120
5.2	118	260	143	121
5.2	158	200	41	26
5.3	7	26	18	254
5.3	24	50	26	110
5.5	18	68	50	277
5.5	20	93	73	367
5.7	11	59	49	460
5.7	101	153	53	52
5.7	244	286	42	17
5.9	12	84	72	578
5.9	13	35	22	177
6.1	5	30	25	491
6.2	1	37	37	5883
6.2	2	66	64	3490
6.2	29	84	56	195
6.3	192	270	78	41
6.4	8	145	138	1832
6.5	18	82	64	347
6.6	4	51	47	1261
6.6	304	354	51	17
6.6	37	160	123	332
6.6	39	178	139	355
7.7	43	105	63	148
9.0	16	49	32	196
10.8	150	243	93	62
12.3	58	129	72	125
12.7	2	43	41	1719
12.8	2	28	26	1211
13.4	130	246	117	90
14.4	6	49	43	684

All volumes are in ml; *DWI* = diffusion weighted images.

No significant difference was found between the retrospective and prospective studies with respect to DWI size and mismatch percentage. There is a significant difference in mismatch volume (smaller in the prospective study), likely due to difference in perfusion analysis techniques. The main hypothesis is validated in both groups.

## Discussion

The occlusion of the terminal ICA and/or proximal MCA accounts for the majority of deaths and poor outcomes in patients with acute ischemic stroke [13,14]. Outcomes may be improved by treatment, but it is important to identify those who are most likely to benefit. In recent years it has been hypothesized that such patients may be identified using physiological information provided by imaging, specifically patients with a major diffusion/perfusion mismatch [10,11]. We have found in retrospective and prospective investigations that every acute stroke patient with terminal ICA and/or proximal MCA occlusion had a major (>100%) diffusion/perfusion mismatch when the DWI lesion was 70 ml or less. If verified, our findings suggest a simple alternative to perfusion imaging based solely on vessel imaging and diffusion MRI to identify those patients with major anterior circulation strokes that are most likely to benefit from treatment.

The use of advanced neuroimaging to identify patients most likely to benefit from therapy has been increasing in recent years. Diffusion MRI is used to estimate the core infarct, and perfusion-weighted imaging is used to delineate hypoperfused tissue. The diffusion/perfusion mismatch is considered the operational penumbra. This approach has been successfully used in treating patients with IV tPA (Tissue plasminogen activator) outside the 3 hour limit [15]. In the DEFUSE Trial [10], it was shown that the use of tPA after 3 hours was effective if there was a significant diffusion/perfusion mismatch that included a DWI lesion of less than 100 ml. It has also been used as inclusion criteria in successful phase 2 trials of desmoteplase, DIAS and DEDAS [10,11], in which patients were given the drug outside the 3 hour limit.

However, recent data including a meta-analysis of the mismatch trials have raised questions regarding the effectiveness of the mismatch approach [2,16]. In parallel, there has been increasing concern over the reliability of perfusion imaging data [17]. Quantification of cerebral hemodynamics is affected by numerous factors which have not been standardized, including image acquisition, post-processing and choice of perfusion parameter [4-6]. Furthermore, one of the usual measures used, the percentage of mismatch, ignores the volume. A 5 ml infarct and a 10 ml region of underperfusion would yield the same percentage of mismatch as a 50 ml infarct and a 100 ml region of underperfusion. Additional problems with perfusion methods include the time required for data acquisition and processing, and the risks related to further contrast administration. Most current approaches for post-processing of the perfusion data require its transfer to a dedicated workstation for further analysis, and a trained individual to process it.

Our observation that a proximal anterior circulation artery occlusion with a DWI lesion of 70 ml or less predicts a diffusion/perfusion mismatch of at least 100% suggests a simple alternative to perfusion methods for identifying major stroke patients most likely to benefit from treatment. This observation is logical considering the relevant physiology. Occlusion of the distal ICA and/or the proximal MCA puts at risk a large brain region that measures well over 200 ml [18]. The state of the brain at the time of evaluation depends on the vigour of the collateral flow, which determines the sizes of the infarct core and penumbra reflected in the diffusion/perfusion mismatch. The collateral flow that arises after ICA/MCA occlusion makes the core and penumbra interdependent parameters [19,20]. Excellent collateral flow accompanying a major occlusion will result in a small diffusion abnormality and a large mismatch (Figure 1), with the opposite result in the setting of poor collateral flow (Figure 2).

We are not advocating a 100% mismatch for clinical decisions, only that in this subset of patients a large mismatch (definitely >20% and usually much larger) is almost certainly present and clinicians can reliably use this information to manage patients.

Our choice of the diffusion abnormality volume threshold of 70 ml was based on previous observations that patients with anterior circulation occlusions and diffusion abnormalities larger than 70 ml have poor outcomes regardless of treatment [21-24], including successful endovascular recanalization [19]. Patients with small diffusion abnormalities had good outcomes only if the occluded arteries were promptly recanalized [19]. There may be a more optimal DWI threshold, and this is an area that merits further study. The measurement of the abnormal DWI volume can be performed manually or semi-automatically using a variety of software. Alternatively, it has been demonstrated that DWI or PWI lesion volumes can be reliably approximated using the formula ABC/2, where A, B, and C are the longest 3 orthogonal distances of the lesion as measured on the MR console or PACS workstation [25,26].

It should be emphasized that the method proposed here is restricted to the specific condition of patients with distal ICA and/or mainstem MCA occlusions. Perfusion imaging may remain beneficial and superior in situations where there are occlusions of anterior circulation branch arteries, proximal ICA, and posterior circulation strokes. It is also probable that perfusion imaging will be of benefit in those without an identifiable occlusion, and in many situations in which a TIA is in the differential diagnosis.

## Conclusions

Patients with acute terminal ICA and/or proximal MCA occlusion will have a diffusion/perfusion mismatch at least as large as the DWI abnormality if the DWI lesion volume is 70 ml or less. Thus, identification of such an occlusion, easily made by CT or MR angiography, and the finding of an abnormal DWI volume of less than 70 ml can identify patients most likely to benefit from an intervention as effectively and more simply than identifying a diffusion/perfusion mismatch as is currently practiced.

## Abbreviations

ADC: Apparent diffusion coefficient; AIF: Arterial input function; CTA: Computed tomography angiography; DWI: Diffusion weighted images; ED: Emergency department; HIPAA: Health insurance portability and accountability; HU: Hounsfield unit; ICA: Internal carotid artery; IRB: Institutional review board; MCA: Middle cerebral artery; MRA: Magnetic resonance angiography; MTT: Mean transit time; PACS: Picture archiving and communication systems; PWI: Perfusion weighted imaging; TE: Echo time; Tmax: Time to maximum; tPA: Tissue plasminogen activator; TR: Repetition time; TTP: Time to peak.

## Competing interests

The authors declare that they have no competing interests.

## Authors' contributions

RH performed image analysis, analyzed and interpreted the data and prepared the manuscript. AJY analyzed and interpreted the data, and prepared the manuscript. JH assisted with data collection and analysis. LHS and ML provided critical review of the manuscript. PWS performed image analysis and provided critical review of the manuscript. RGG conceived the study, analyzed and interpreted the data and prepared the manuscript. All authors read and approved the manuscript.

## Pre-publication history

The pre-publication history for this paper can be accessed here:

http://www.biomedcentral.com/1471-2377/12/132/prepub
